# The effect of NAMPT deletion in projection neurons on the function and structure of neuromuscular junction (NMJ) in mice

**DOI:** 10.1038/s41598-019-57085-4

**Published:** 2020-01-09

**Authors:** Samuel Lundt, Nannan Zhang, Xiaowan Wang, Luis Polo-Parada, Shinghua Ding

**Affiliations:** 1 Department of Biomedical, Biological and Chemical Engineering, University of Missouri, Columbia, MO 65211 USA; 2 Dalton Cardiovascular Research Center, University of Missouri, Columbia, MO 65211 USA; 3 Department of Medical Pharmacology and Physiology, University of Missouri, Columbia, MO 65211 USA

**Keywords:** Synaptic vesicle endocytosis, Neurophysiology

## Abstract

Nicotinamide adenine dinucleotide (NAD^+^) plays a critical role in energy metabolism and bioenergetic homeostasis. Most NAD^+^ in mammalian cells is synthesized via the NAD^+^ salvage pathway, where nicotinamide phosphoribosyltransferase (NAMPT) is the rate-limiting enzyme, converting nicotinamide into nicotinamide mononucleotide (NMN). Using a Thy1-Nampt^−/−^ projection neuron conditional knockout (cKO) mouse, we studied the impact of NAMPT on synaptic vesicle cycling in the neuromuscular junction (NMJ), end-plate structure of NMJs and muscle contractility of semitendinosus muscles. Loss of NAMPT impaired synaptic vesicle endocytosis/exocytosis in the NMJs. The cKO mice also had motor endplates with significantly reduced area and thickness. When the cKO mice were treated with NMN, vesicle endocytosis/exocytosis was improved and endplate morphology was restored. Electrical stimulation induced muscle contraction was significantly impacted in the cKO mice in a frequency dependent manner. The cKO mice were unresponsive to high frequency stimulation (100 Hz), while the NMN-treated cKO mice responded similarly to the control mice. Transmission electron microscopy (TEM) revealed sarcomere misalignment and changes to mitochondrial morphology in the cKO mice, with NMN treatment restoring sarcomere alignment but not mitochondrial morphology. This study demonstrates that neuronal NAMPT is important for pre-/post-synaptic NMJ function, and maintaining skeletal muscular function and structure.

## Introduction

Nicotinamide adenine dinucleotide (NAD^+^) is found in all cells of the human body and is an important cofactor or co-substrate, used in numerous enzymatic processes including glycolysis, the tricarboxylic acid (TCA) cycle, oxidative phosphorylation, DNA repair, and protein deacetylation^1^. NAD^+^ levels may be important for many aspects of health and aging, including cellular metabolism, sarcopenia, and neurodegeneration^2^. NAD^+^ can be synthesized through multiple enzymatic pathways. One is a *de novo* pathway that begins with the amino acid tryptophan, while other pathways utilize different metabolites capable of being converted into NAD^+^. In mammalian cells, the majority of NAD^+^ is produced from metabolites entering the NAD^+^ salvage pathway^3^. The rate limiting enzyme of the salvage pathway is nicotinamide phosphoribosyltransferase (NAMPT), which condenses nicotinamide (NAM) and 5-phosphoribosyl pyrophosphate (PRPP) into nicotinamide mononucleotide (NMN). NMN is subsequently synthesized into NAD^+^ by nicotinamide mononucleotide adenylyltransferases (NMNATs)^4^.

NAD^+^ levels decline with age and in different diseases. However, administration of NAD^+^ precursor molecules, such as NMN or nicotinamide riboside (NR), are effective at preventing or reversing many age- or disease-related declines^5–10^. NAD^+^ and the NAD^+^ salvage pathway are vitally important to maintain bioenergetic homeostasis, the normal health and function of many different organs and tissues in the human body, with neurons and skeletal muscles being impacted greatly. In neuronal cell cultures, increased NMNAT activity or NAD^+^ pre-treatment could prevent axon degeneration following physical or chemical injury^11^. Overexpression of NAMPT is also able to delay axonal degeneration following axotomy^12^ and can elevate NAD^+^ levels in neurons^13^. The protective effects of NAD^+^ on neurons have not only been shown *in vitro*, mice administered NMN following an ischemic event had reduced neuronal death and prevented cognitive and motor impairments^14^. Knockout of NAMPT from Schwann cells, cells responsible for myelinating and providing metabolic support to peripheral axons, produces severe peripheral neuropathy and death but these problems can be attenuated when an NAD^+^ precursor is provided^15^. There is also evidence indicating a possible therapeutic role for NAD^+^. P7C3, an NAMPT enhancing compound, has been shown to be neuroprotective in stroke^16^, Parkinson disease^17^, and amyotrophic lateral sclerosis (ALS) rodent models^18^.

Skeletal muscle is also affected by changes to NAD^+^ biosynthesis. Energy production of skeletal myotubes is impaired following NAMPT inhibition^19^. Deletion of NAMPT from the tibialis anterior muscle in mice reduced NAD^+^ availability and mitochondrial function^20^. Mice lacking NAMPT in skeletal muscles exhibited many impairments, including reduced muscle mass, twitch force, and respiratory capacity but those problems were reversed when those mice were provided NR^9^. Mice over-expressing NAMPT in skeletal muscles had elevated NAD^+^ and metabolite levels and showed improved exercise endurance capacity when allowed voluntary exercise^21^. NAD^+^ supplementation has also been shown to be beneficial to skeletal muscle disorders. In mdx mice, a model of Duchene’s muscular dystrophy, NR supplementation was able to reduce fibrosis and improve skeletal muscle function^7^. How neurons and skeletal muscles are individually affected by altering NAD^+^ homeostasis has been well studied but how disruptions of the neuronal NAD^+^ salvage pathway may impact the neuromuscular junction (NMJ), where nerves and muscles interact, is less understood.

Our recent study showed that the loss of NAMPT in projection neurons, using a Thy1-Nampt^−/−^ conditional knockout (cKO) mouse model, produced neurodegeneration, muscle atrophy, abnormal NMJs and, eventually, death. These symptoms are similar to those observed in ALS. When these cKO mice were administered with NMN, these detrimental effects were ameliorated and lifespan was extended^22^. These results imply a non-cell autonomous effect of neuronal NAMPT on skeletal muscle structure and function. In current study, using the same cKO mouse model, we further investigated the effects of neuronal NAMPT on the structure and function of NMJs and skeletal muscles. We found that deletion of Nampt disrupted the normal synaptic vesicle cycling, lead to morphological changes to the motor endplate alteration of muscle contractility and sarcomere misalignment. We also found that treatment of NAD^+^ precursor NMN had beneficial effects on vesicle cycling, endplate morphology and muscle contractility of the cKO mice. Our study suggests that the disruption of bioenergetic homeostasis in projection neurons could significantly affect synaptic vesicle cycling at NMJs and muscular function and structure.

## Materials and Methods

### Mice

Mice were maintained on a 12 h light:12 h dark cycle (lights on 7 am–7 pm) in our AAALAC-accredited animal facility at the University of Missouri. All experimental procedures were performed in accordance with the NIH Guide for the Care and Use of Laboratory Animals and approved by the University of Missouri Animal Care Quality Assurance Committee (#9444). Both male and female adult mice were used for this study. Generation of projection neuron-specific conditional knockout (*Thy1-YFP-Nampt*^−/−^) mice was done following the same procedure as described in our previous publication^22^. Briefly, *Thy1-CreER*^*T2*^*-YFP* mice (Jackson Laboratory)^23^ were crossed with *Nampt*^*f/f*^ mice^24^ to obtain *Thy1-CreER*^*T2*^*-YFP: Nampt*^*f/f*^ double homozygous transgenic mice. Nampt was deleted by administration of tamoxifen (TAM), dissolved in sunflower oil, with a dose of 200 mg/kg bodyweight, via oral gavage, for 5 consecutive days. We designated *Thy1-YFP-Nampt*^−/−^ mice for homozygous Nampt^−/−^ cKO mice. Starting 10 days after the final TAM administration, *Thy1-YFP-Nampt*^−/−^ mice were given a daily intraperitoneal injection of either 0.9% saline solution or NMN solution, with a dose of 400 mg/kg. All NMN-treated mice were administered NMN daily for at least 14 days prior to sacrifice. Body weights were recorded at the same time daily.

### Imaging of vesicle cycling with FM1-43 dye

Vesicle cycling in semitendinosus muscle was studied by imaging change of FM1-43 fluorescence. FM1-43 is a styryl dye that capable of labeling synaptic vesicles undergoing endocytosis and exocytosis^25^. For FM1-43 imaging of semitendinosus muscle, mice were sacrificed between 21 and 28 days after the last TAM administration, and the muscles were rapidly isolated, taking care to leave the nerve attached intact. The muscles were placed in Tyrode’s solution (140 mM NaCl, 5.6 mM KCl,1 mM MgCl_2_, 2 mM CaCl_2_, 1.8 mM Na_2_HPO_4_, 10 mM NaHCO_3_, 5.5 mM glucose) receiving 95%O_2_/5%CO_2_ continuously. Excessive non-semitendinosus muscle, connective tissue, and fat tissue were removed from the muscles and electrical stimulation was applied to the nerve to ensure the muscle was contracting. Clean muscles were pinned flat in a recording dish with sylgard bottom and filled with Tyrode’s solution receiving 95%O_2_/5%CO_2_ continuously.

The attached nerve was stimulated using a suction electrode to find a minimum current for muscle contraction. Muscle was incubated with Alexa-555 conjugated α-bungarotoxin (α-BTX-555) (1.5:1000, Cat. No. B35451, Invitrogen) to block contractions. After contractions stopped, the muscle was stimulated at twice the minimum current threshold (10 Hz, 10 ms delay, 6 ms duration; Grass S88 Stimulator). Time-lapse imaging of vesicle endocytosis was conducted with a speed of one frame/minute, for 30 minutes, in Tyrode’s solution containing 12 μM FM1-43 fluorescent dye (Cat. No. F35355, Life Technologies). The muscle was then washed twice with low Ca^2+^ Tyrode’s solution (140 mM NaCl, 5.6 mM KCl, 5 mM MgCl_2_, 0.2 mM CaCl_2_, 1.8 mM Na_2_HPO_4_, 10 mM NaHCO_3_, 5.5 mM glucose) 5 minutes for each time. The first wash contained 10 μM ADVASEP^TM^-7 (A3723, Sigma-Aldrich) to reduce background fluorescence. Time-lapse imaging for vesicle exocytosis was conducted with a speed of one frame/minute, for 25 minutes, while muscle was stimulated in Tyrode’s solution.

Imaging was performed using a Nikon Eclipse FN1 fluorescent microscope with a 40x water immerse Olympus objective (LUMPlanFI/IR, NA/0.8). Images were acquired as stack files using a Photometric Cool SNAP EZ CCD camera controlled by Metaview software. Image analysis was performed using Metamorph software. Acquired stack images were aligned, and a region-of-interest was assigned to assess fluorescent intensity changes of the neuromuscular junction.

For imaging FM1-43 release for an extended period of time, all procedures were identical except for the length of the second stimulation period. The second stimulation time was increased from 25 minutes to 120 minutes. For the spontaneous FM1-43 uptake, all procedures were identical except no stimulation occurred. Following the incubation with FM1-43 for 30 minutes, the muscles used for spontaneous FM1-43 uptake were fixed with 3.7% paraformaldehyde and stored at −20 °C until the NMJs were imaged.

### Labeling and structural analysis of motor endplate

Isolated clean semitendinosus muscles were incubated with α-BTX-555 (1.5:1000) for 10–15 minutes, and then washed 3 times for 10 minutes each. Muscles were then covered with OCT compound and flash frozen in liquid nitrogen. Muscles were stored at −20 °C prior to cutting. Using a cryostat (Leica CM1900), cross-sections of semitendinosus muscles were cut onto coated microscope glass slides with a thickness of 5 μm. Muscle slices were carefully washed with distilled water to remove OCT. Motor endplate images were taken using the same fluorescent microscope and camera as the FM1-43 imaging but with a lens of 60x Olympus objective (A 60, NA/0.80). Measurements of area, length, breadth, outer radius, inner radius, and average intensity were performed by Metamorph software. Depression depth, depression width, left thickness, right thickness, and bottom thickness were manually measured using the Single Line function in Metamorph.

### Measurement of muscle contractile response

Mice were sacrificed between 21 and 28 days after the last TAM injection. Semitendinosus isolation procedure was the same as the FM1-43 staining. Muscle was pinned to a sylgard dish, with the wide end fixed with a pin and the narrow end left free. A piece of string was used to connect the muscle to a force transducer (MLT500/A, ADInstruments). The string was looped around the narrow end of the muscle and pulled tight. The force transducer was moved parallel to and away from the muscle until the muscle was pulled tight. The force transducer was connected to an amplifier (PowerLab 4/25 T, ADInstruments). The muscle was stimulated at 1, 5, 10, 20, 50, and 100 Hz using a Grass SD9 stimulator. Responses were recorded using LabChart software.

Muscle contraction parameters were assessed using LabChart software. Rise time, *i.e*., the time from the onset of the response after stimulation to the maximal response of contraction force. Amplitude was the difference between baseline and maximum contraction force. Fall time was the amount of time it took to return to baseline once stimulation was removed. Slope was calculated as the amount of force lost from when maximum contractile force was reached until the point stimulation was ended, divided by the length of time from maximum contractile force until stimulation was removed (grams of force loss/second).

### NAD^+^ assay of semitendinosus muscle

The contents of NAD^+^ were measured using a commercially available assay kit (E2ND-100, Bioassay Systems, CA)^22,26^. To study the effect of NMN on NAD^+^ levels in muscles, NMN was administered to mice via intraperitoneal injection for two consecutive days, with the mice being sacrificed 1 hour after the second injection. Non-injected mice were used as controls. Semitendinosus and gastrocnemius muscles were dissected from mice, placed into a 1.5 mL tube with 100 μL NAD^+^ extraction buffer, and homogenized on ice. The extracts were heated at 60 °C for 5 min, and then 20 μL assay buffer and 100 μL of the opposite extraction buffer were added to neutralize the extracts. The samples were briefly mixed and spun down at 14,000 rpm for 5 minutes. The supernatant was used for NAD^+^ assay. The total NAD^+^ contents were expressed as pmol/mg tissue.

### Western blotting analysis

Western blotting was used to analyze NAMPT in semitendinosus muscles as described in our previous studies^22,27–31^. Briefly, the fresh muscle tissues were dissolved in lysis buffer mixed with protease inhibitor (Cat. No. 36978, Pierce Biotechnology, Rockford, IL) and cocktails of phosphatase inhibitor (Cat. No. P8340 Sigma-Aldrich), and homogenized. The homogenized tissues were centrifuged at 13,500 g for 30 minutes at 4 °C, and the total lysate was kept in the supernatant. The protein concentration of the cell lysate was measured by bicinchoninic acid (BCA) protein assay kit (Cat. No. 23277, Pierce Biotechnology). The protein samples were boiled for 5 minutes, loaded in 10% SDS-polyacrylamide gels, subjected to electrophoresis at 100 mV for 100–110 minutes, and transferred to polyvinylidene fluoride membranes using Mixed Molecular Weight function of Trans-Blot Turbo Transfer System (Bio-Rad, Hercules, CA). The membranes were blocked for 1 hour by 5% (w/v) bovine serum albumin (BSA) in Tris-buffered saline containing 0.1% (v/v) Tween-20 (TBS-T), and were then incubated overnight at 4 °C with mouse monoclonal anti-NAMPT (1:1000, ALX-804-922-C100, Enzo Life Sciences, Farmingdale, NY) in 1% (w/v) BSA. After washing with TBS-T, the membranes were incubated with the second antibodies HRP (horseradish peroxidase)-conjugated rabbit anti-mouse IgG (1:2500, A9044, Sigma-Aldrich) in 1% (w/v) BSA for 1 hour at room temperature. Monoclonal mouse anti-β-actin antibody (1:1000, cs-47778, Santa Cruz, CA) was used as a control of equal loading for the total protein in cell lysate. Precision Plus Protein™ Dual Color Standards (Cat. No. 161-0374, Bio-Rad) was used as the marker to evaluate the molecular size of protein bands. Membranes were exposed to Clarity™ Western ECL Substrate (Cat. No. 170-5061, Bio-Rad) and imaged with ChemiDoc™ XRS + system (Bio-Rad).

### Transmission electron microscopy (TEM)

Semitendinosus muscles were isolated from Nampt^−/−^ cKO, NMN-treated Nampt^−/−^ cKO, and Nampt^f/f^ mice 23 days after the final TAM administration. The muscles were cleaned of excessive non-semitendinosus muscle, connective tissue, and fat. The muscles were cut into smaller sections, with only the sections that likely contained NMJs retained. Remaining sections were fixed in 100 mM sodium cacodylate buffer containing 2% paraformaldehyde and 2% glutaraldehyde (pH 7.35). Samples were left at room temperature for at least 1 hour and moved to 4 °C for at least 23 hours. Fixed samples were washed with 100 mM sodium cacodylate buffer (pH 7.35) containing 130 mM sucrose. Secondary fixation was performed using 1% osmium tetroxide in cacodylate buffer using a Pelco Biowave (Ted Pella, Inc. Redding, California) operated at 100 Watts for 1 minute. Samples were incubated at 4 °C for 1 hour, then rinsed with cacodylate buffer, followed with distilled water. En bloc staining was performed using 1% aqueous uranyl acetate and incubated at 4 °C overnight, then rinsed with distilled water. A graded dehydration series (30%, 50%, 70%, 90%, 100%, 100%) was performed using ethanol at 4 °C, transitioned to acetone. Dehydrated samples were then infiltrated with Epon resin for 24 hours at room temperature and polymerized at 60 °C for 48–72 hours. Samples were cut into 85 nm thick longitudinal and transverse sections using an ultramicrotome (Ultracut UCT, Leica Microsystems, Germany) and a diamond knife (Diatome, Hatfield, PA). 2000X and 6000X images were acquired with a JEOL JEM 1400 transmission electron microscope (JEOL, Peabody, MA) at 80 kV (0.35 s exposure time) on a Gatan Ultrascan 1000 CCD (Gatan, Inc, Pleasanton, CA). Myofiber composition and mitochondria morphology (area, perimeter, circularity, and Feret’s diameter) were assessed using ImageJ (NIH). Circularity is scored from 0 to 1, with a score of 1 reflecting a perfect circle and the score decreasing as the shape become elongated. Feret’s diameter is the longest distance between any two points along the outlined area and can be referred to as caliper distance. These parameters have been used previous to characterize mitochondrial morphology in skeletal muscle^32^.

### Statistical analysis

Data expressed as mean ± standard error of the mean (SEM). Comparisons were made using unpaired Student’s t-test or One-way ANOVA with Tukey post-test. Levels of significance set as *p < 0.05, **p < 0.01, and ***p < 0.001. Statistical analyses were performed using OriginPro 8 and Excel.

## Results

### Loss of NAMPT in projection neurons impairs synaptic endocytosis and exocytosis at NMJs

**Figure 1:**
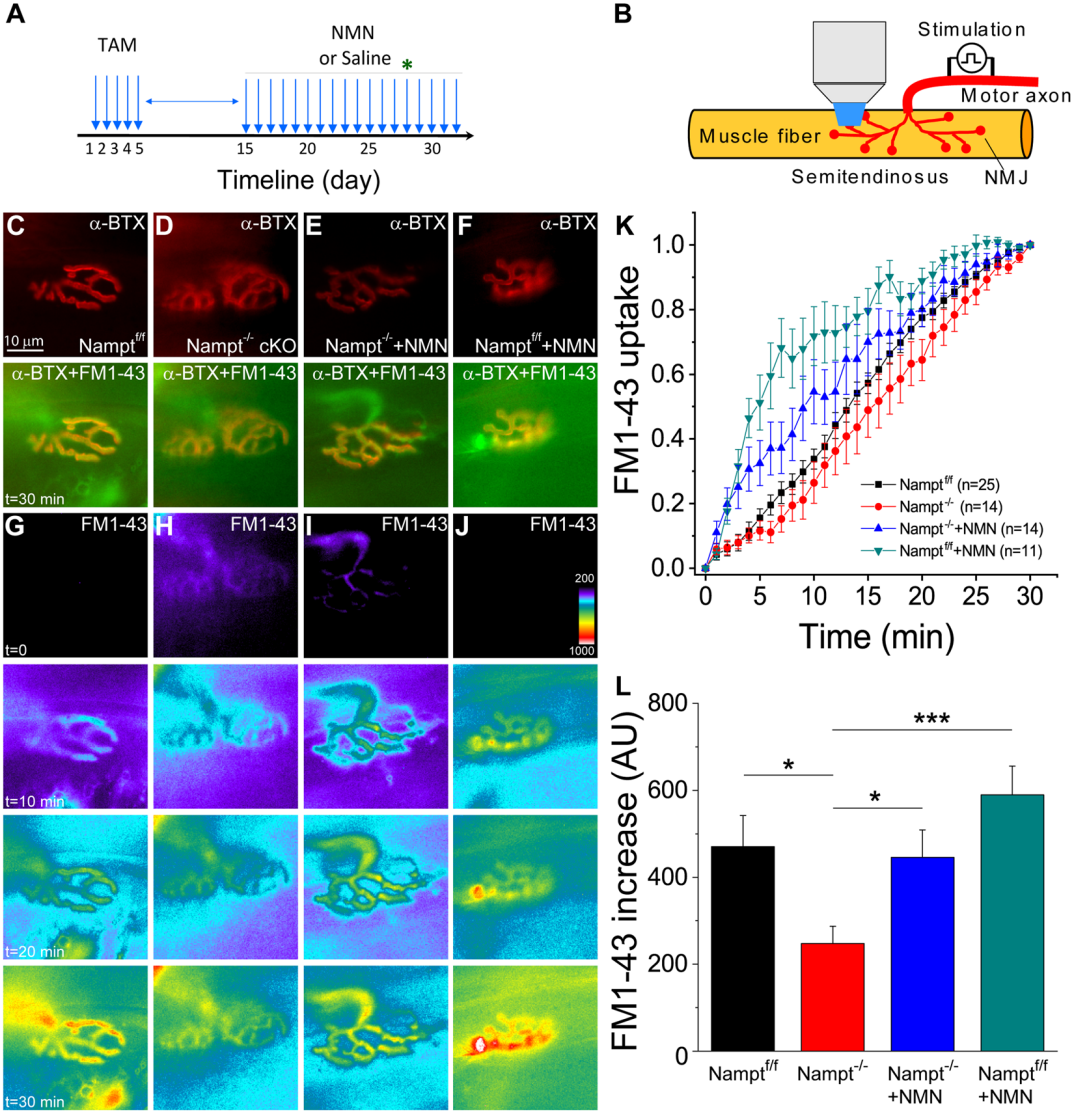
Impaired endocytosis in motor nerve terminal of Nampt^−/−^ cKO mice. **(A)** Experimental timeline. Star indicates the starting date of imaging. **(B)** Illustration of FM1-43 imaging of NMJs. Motor axon is stimulated for staining and destaining of FM1-43. (**C–F**) Motor endplates of Nampt^f/f^ mice (**C**), Nampt^−/−^ cKO mice (**D**), NMN-treated Nampt^−/−^ cKO mice **(E**), and NMN-treated Nampt^f/f^ mice (**F**). The top row shows images of nAChRs in NMJs labeled with α-BTX conjugated with Alexa 555, and the 2nd row shows the merged images of the same NMJs labeled with α-BTX and FM1-43. Nerve terminals were stained with FM1-43 dye and images were taken at speed of one frame/minute after 30 minutes of stimulation with 10 Hz in the presence of the dye. (**G–J**) Representative fluorescent images of FM1-43 uptake in nerve terminal before stimulation, and 10, 20 and 30 minutes after stimulation. (**K**) Normalized intensity of FM1-43 uptake in nerve terminals. Images were taken at speed of one frame/minute during 30 min stimulation with 10 Hz in the presence of the FM1-43 dye. **(L**) Mean fluorescence increase in nerve terminal for Nampt^f/f^ (470.5 ± 71.5, n = 24), Nampt^−/−^ cKO (247.4 ± 40.0, n = 16), NMN-treated Nampt^−/−^ cKO (445.8 ± 63.1, n = 13). Mice, and NMN-treated Nampt^f/f^ mice (589.2 ± 66.6, n = 11). Mean pixel intensity was calculated after 30 minutes of stimulation. n is the number of motor nerve terminals from 11–21 mice. *p < 0.05, ***p < 0.001, Student’s t-test.

Our previous study has shown that Nampt deletion in projection neurons of mice produced NMJ abnormalities and reduced synaptic transmission^22^. To further understand the phenomena induced by Nampt deletion, using FM1-43 imaging, we investigated if the endocytosis and exocytosis of synaptic vesicles, important for synaptic function, at the NMJs have also been changed in Nampt^−/−^ cKO mice. We isolated the semitendinosus muscles from Nampt^f/f^ control mice and Nampt^−/−^ cKO mice treated for two weeks with either saline or NMN (400 mg/kg dose). We also isolated the semitendinosus muscles from Nampt^f/f^ mice treated with NMN for 2 weeks as an additional control. Figure 1A,B indicates the experimental timeline and illustration of FM1-43 imaging of NMJs. To study endocytosis and exocytosis, the isolated muscles were incubated with a fluorescent α-BTX to block contractions and loaded with FM1-43 for imaging of synaptic vesicle endocytosis (Fig. 1C–F). Once loaded with FM1-43, the muscles underwent a second period of stimulation without FM1-43 in the extracellular solution. Time lapse imaging was conducted to track changes in FM1-43 fluorescence to study endocytosis and exocytosis in the same NMJs.

Figure 1G,J shows fluorescent images of the NMJs at different times during stimulation with FM1-43. For all four conditions, the normalized FM1-43 fluorescence increased in the motor nerve terminals, indicating FM1-43 uptake (Fig. 1K). Our data show that Nampt^−/−^ cKO mice had a slower FM1-43 uptake rate than Nampt^f/f^ mice, while NMN-treatment could increase FM1-43 uptake in Nampt^−/−^ cKO mice; NMN-treated Nampt^f/f^ mice exhibit the fastest FM1-43 uptake rate. The total FM1-43 fluorescence uptake in Nampt^−/−^ cKO mice (247.4 ± 40.0 a.u.) following 30 minutes of stimulation, was significantly reduced as compared with Nampt^f/f^ mice (470.5 ± 71.5 a.u.) (Fig. 1L). Treatment of Nampt^−/−^ cKO mice with NMN significantly increased total FM1-43 fluorescence (445.8 ± 63.1 a.u.) to a value similar to Nampt^f/f^ mice, while treatment of Nampt^f/f^ mice with NMN did not significantly increase in FM1-43 fluorescence (589.2 ± 66.6 a.u.) (Fig. 1H). These results suggest that loss of NAMPT reduces the rate and amount of evoked endocytosis, while treatment of NMN can restore endocytosis.

**Figure 2:**
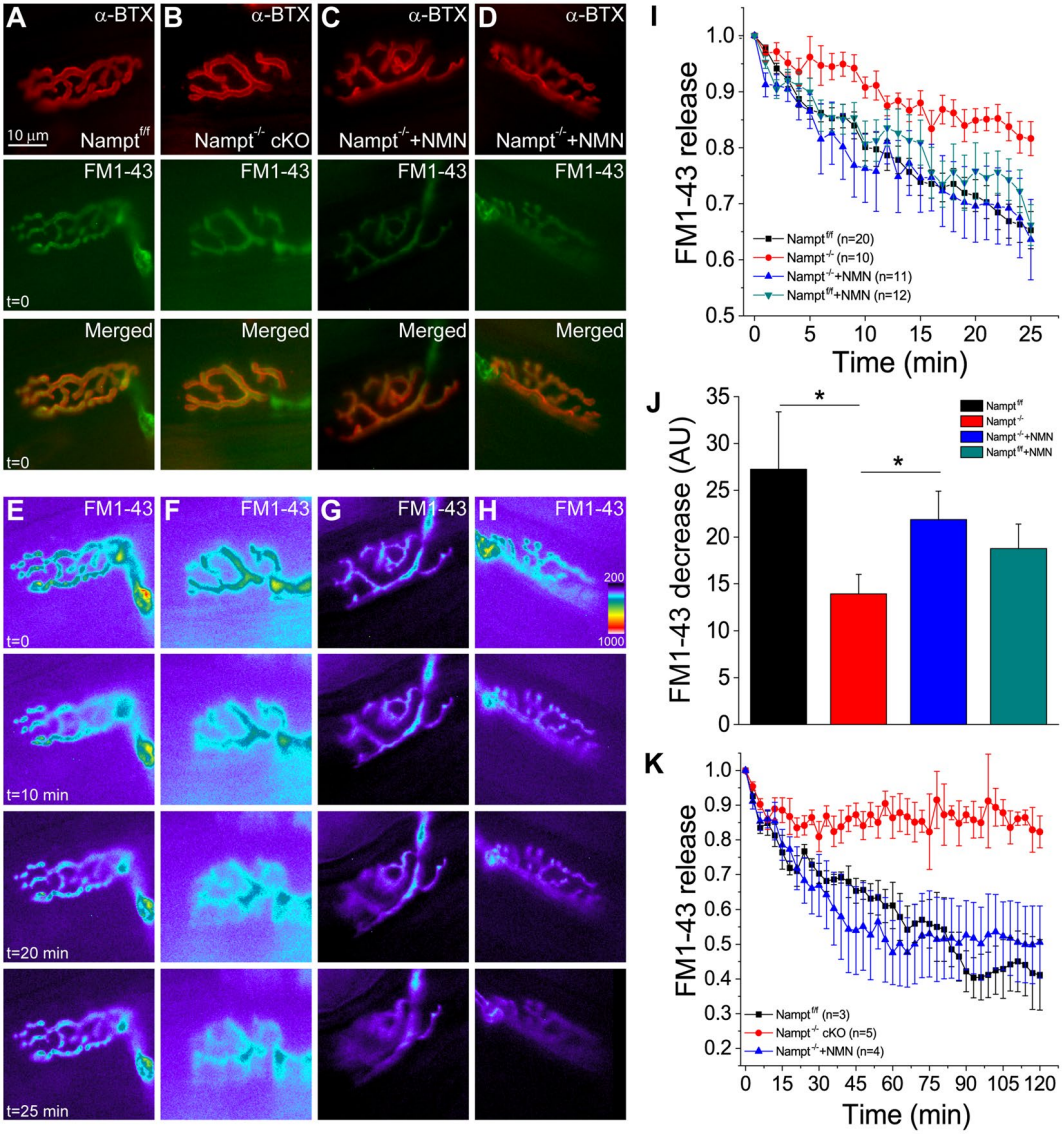
Impaired exocytosis from motor nerve terminal in Nampt^−/−^ cKO mice. **(A–D**) Motor endplates of Nampt^f/f^ mice (**A**), Nampt^−/−^ cKO and NMN-treated Nampt^−/−^ cKO mice (**B,C**), and NMN-treated Nampt^f/f^ mice (**D**). nAChRs were labeled with α-BTX conjugated with Alexa 555 (top row). Nerve terminal of the corresponding motor endplate in (**A–D**) stained with FM1-43 prior to destain stimulation (row second from top). Images were taken prior to stimulation. Merged images of the NMJs in (**A–D**) labeled with α-BTX and FM1-43 (row third from top). (**E–H**) Fluorescent imaging of FM1-43 release from nerve terminal before stimulation, and 10, 20 and 25 minutes after stimulation. (**I**) Normalized intensity of FM1-43 release from nerve terminal. Images taken every minute during the 25 minutes of stimulation. **(J**) Mean fluorescence decrease from nerve terminal for Nampt^f/f^ (27.2 ± 6.1, n = 9), Nampt^−/−^ cKO (14.0 ± 2.0, n = 11), and NMN-treated Nampt^−/−^ cKO (21.9 ± 3.0, n = 10), and NMN-treated NAMPT^f/f^ mice (18.8 ± 2.6, n = 12). Mean pixel intensities were calculated after 25 minutes of stimulation. *p < 0.05, Student’s t-test. (**K**) Normalized intensity of FM1-43 release from nerve terminal. Image taken every three minutes during the 120 minutes of stimulation. n reflects the number of motor nerve terminals analyzed from 8–18 (**J**) and 3–4 (**K**) mice.

Next, we studied the effect of Nampt deletion on exocytosis. Time-lapse imaging was conducted for 25 minutes with stimulation of nerve-muscle preparations that had been successfully loaded with FM1-43 in the previous stimulation period (Fig. 2A–D). All four conditions showed a reduction in FM1-43 fluorescence within the nerve terminal after stimulation (Fig. 2E–H). The loss of NAMPT lead to a noticeable reduction in the rate of FM1-43 release, while NMN-treated Nampt^−/−^ cKO mice had a similar rate of FM1-43 reduction to Nampt^f/f^ mice treated with and without NMN (Fig. 2I). The total fluorescence reduction was also significantly lower in the Nampt^−/−^ cKO mice (14.0 ± 2.0 a.u.) compared to Nampt^f/f^ mice (27.2 ± 6.1 a.u.), while NMN-treatment restored exocytosis in Nampt^−/−^ cKO (21.9 ± 3.0 a.u.) mice (Fig. 2J). FM1-43 fluorescence reduction in Nampt^f/f^, NMN-treated Nampt^f/f^ (18.8 ± 2.6 a.u.) and NMN-treated Nampt^−/−^ cKO mice were not significantly different (Fig. 2J). These results indicate that loss of NAMPT in projection neurons significantly impairs evoked exocytosis, both in the rate and amount of vesicle release at NMJs, but NMN can largely prevent these impairments.

The differences in the FM1-43 fluorescence reduction after destaining stimulation led us to investigate why this impairment may be occurring. Specifically, whether the rate of FM1-43 release was simply reduced in the Nampt^−/−^ cKO mice or whether there was a problem in accessing a portion of FM1-43 loaded vesicles. To investigate this, we extended the destaining stimulation time from 25 to 120 minutes, while keeping the FM1-43 loading time the same. During the 120 minutes of destaining stimulation, the Nampt^−/−^ cKO mice stopped decreasing in fluorescence around 30 minutes, while the Nampt^f/f^ and NMN-treated Nampt^−/−^ cKO mice continued to decrease until around 90 minutes (Fig. 2K). This suggests that the Nampt^−/−^ cKO mice are unable to release certain vesicles that have been loaded with FM1-43 dye or have a reduced number of synaptic vesicles^22^.

**Figure 3:**
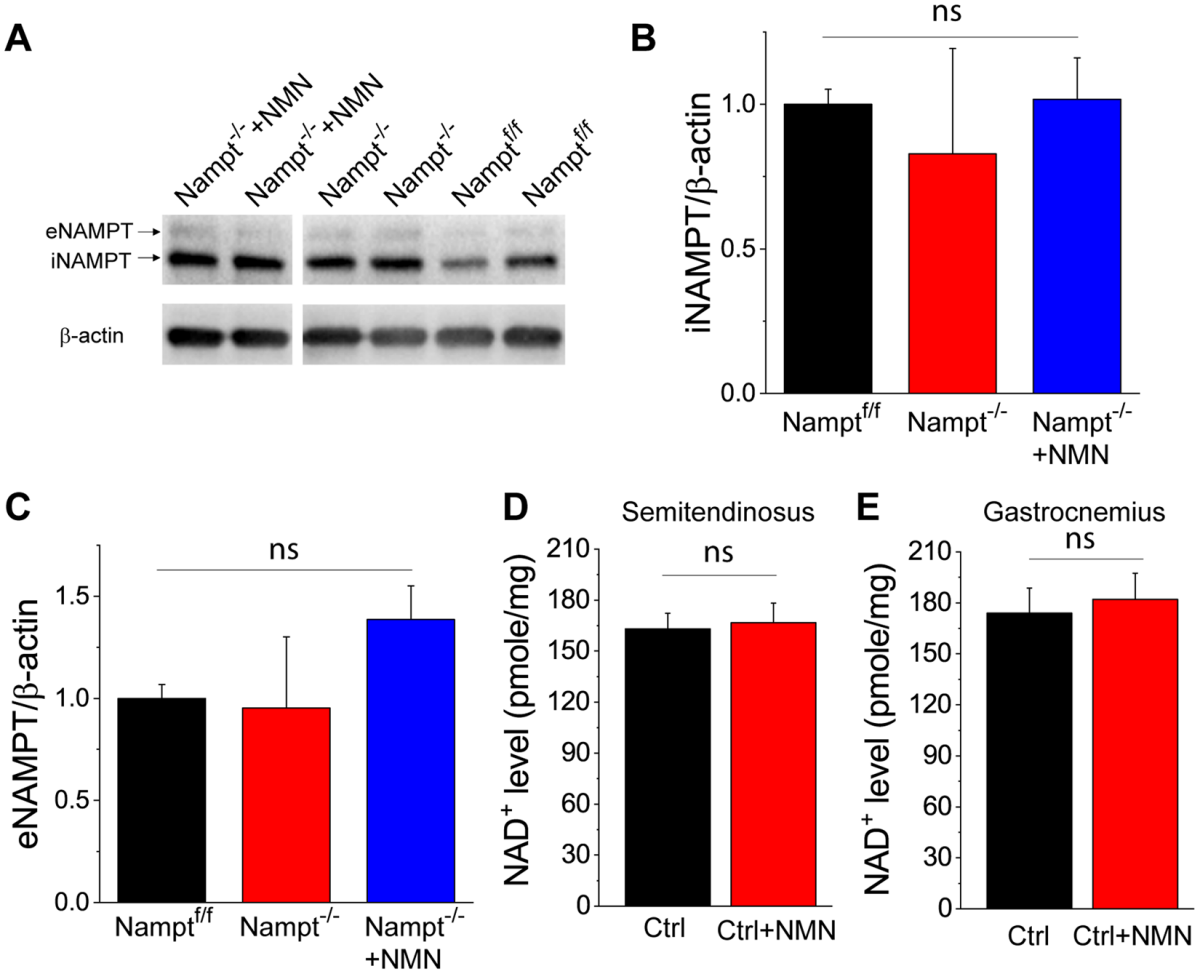
Western blot analysis of iNAMPT and eNAMPT and NAD^+^ assay in the semitendinosus muscles. (**A–C**) Western blot images (**A**) and summary data of iNAMPT (**B**) and eNAMPT (**C**) in the semitendinosus muscles of Nampt^f/f^ mice, Thy1-Nampt^−/−^ cKO mice and NMN-treated Thy1-Nampt^−/−^ cKO mice. (**D,E**) NAD^+^ levels in semitendinosus and gastrocnemius muscles of control mice after NMN administration. Mice were injected NMN (400 mg/kg) for two times 24 hour apart and sacrificed 1 hour after the second injection for NAD^+^ assay of isolated semitendinosus and gastrocnemius muscles. Summary data in (**B–E**) were averaged values from N = 4 mice for each group. ns indicates no significant difference with t-test or ANOVA test. Full-length blots are presented in Supplementary Fig. 1.

Endocytosis and exocytosis are affected by bioenergetics^33,34^. To determine whether the impairments of vesicle recycling is caused by the reduction of NAMPT levels in muscle, we used Western blotting to analyze whether Nampt^−/−^ cKO mice had reduced NAMPT levels in semitendinosus muscles. Our results show that Nampt^−/−^ cKO mice expressed similar intracellular NAMPT (iNAMPT) and extracellular NAMPT (eNAMPT) levels compared to the Nampt^f/f^ control mice, furthermore, NMN administration did not affect iNAMPT and eNAMPT levels in the muscle (Fig. 3A–C). In addition, NMN administration did not increase NAD^+^ levels in the semitendinosus and gastrocnemius muscles of control mice (Fig. 3D–E). These results are consistent with those in our previous study showing that deletion of Nampt in the projection neurons did not affect iNAMPT and eNAMPT levels in non-CNS organs^22^. These results also suggest that altered exocytosis and endocytosis in Nampt^−/−^ cKO mice probably resulted from the long-term effect of neuronal Nampt deletion on NMJs. Since there were no significant changes in endocytosis, exocytosis and NAD^+^ level in muscle of the NMN-treated control mice, we only used three groups of mice, *i.e*., Nampt^f/f^ mice, Nampt^−/−^ cKO mice, and Nampt^−/−^ cKO mice treated with NMN for the rest of study.

**Figure 4:**
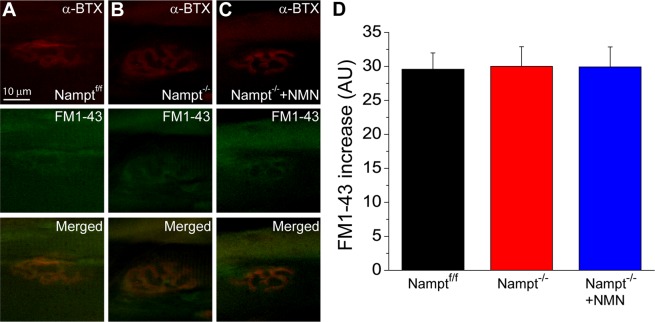
Spontaneous endocytosis in not altered in Nampt^−/−^ cKO mice. **(A–C**) Motor endplate and nerve terminal from Nampt^f/f^ (**A**), Nampt^−/−^ cKO and NMN-treated Nampt^−/−^ cKO mice **(B,C**). nAChRs were labeled with α-BTX conjugated with Alexa 555 (top row) and nerve terminal of the corresponding motor endplate were stained with FM1-43 dye (middle row). Merged images of NMJ from α-BTX and FM1-43 images were shown in bottom row. Images were taken 30 minutes of incubation without nerve stimulation. (**D**) Mean fluorescence increase in nerve terminal of Nampt^f/f^ (29.6 ± 2.4, n = 34), Nampt^−/−^ cKO (30.0 ± 2.9, n = 27), and NMN-treated Nampt^−/−^ cKO (29.9 ± 2.9, n = 36) mice. n is the number of nerve terminals from 3–4 mice.

The aforementioned results of synaptic vesicle cycle imaging provided evidence that the loss of NAMPT in projection neurons was detrimental to the stimulation-induced activity within the synaptic terminals, however, it is unknown whether spontaneous endocytosis is also impacted after Nampt deletion. To assess this, dissected muscles were incubated with FM1-43 for 30 minutes without stimulation, after staining with α-BTX, and then fixed with 4% PFA for imaging of FM1-43 loaded NMJs (Fig. 4A–C). The spontaneous fluorescence increase did not significantly differ among the Nampt^f/f^ (29.6 ± 2.4 a.u.), Nampt^−/−^ cKO (30.0 ± 2.9 a.u.) and NMN-treated Nampt^−/−^ cKO mice (29.9 ± 2.9 a.u.) (Fig. 4D).

### Loss of NAMPT in projection neurons leads to alterations of post-synaptic neuromuscular junction structure

**Figure 5:**
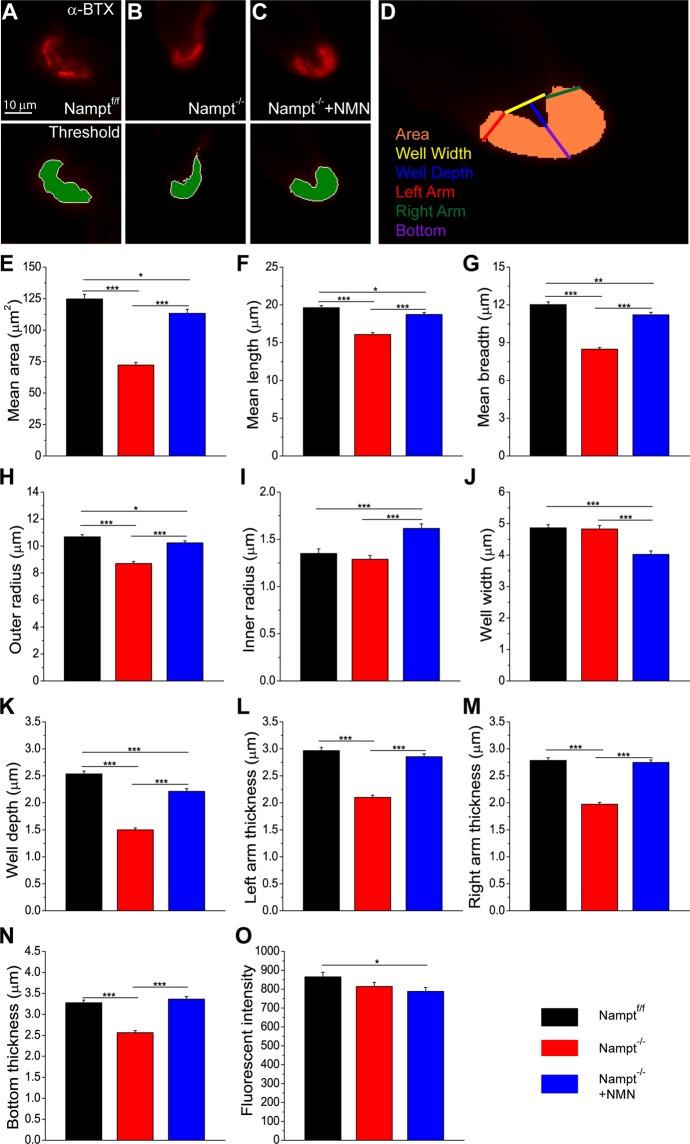
Motor endplate morphology is altered in Nampt^−/−^ cKO mice. (**A–C**) Cross-section of motor endplate from (**A**) Nampt^f/f^ (n = 342), (**B**) Nampt^−/−^ cKO (n = 268) and (**C**) NMN-treated Nampt^−/−^ cKO (n = 290) mice. nAChRs were labeled with α-BTX conjugated with Alexa 555 (top row). Area in green was the region analyzed after an exclusive fluorescence thresholding function was applied to the α-BTX labeled image (bottom row). **D)** Diagram of dimensions manually assessed (well depth, well width, and left, right, and bottom thickness) using the thresholded endplate region. Area was calculated by analysis software. Motor endplate measurements always go Nampt^f/f^, Nampt^−/−^ cKO, and NMN-treated Nampt^−/−^ cKO mice. (**E**) Mean area of motor endplate cross-section; (124.7 ± 3.5), (72.3 ± 2.1), and (113.3 ± 3.1). (**F**) Mean length of motor endplate; (19.6 ± 0.3), (16.1 ± 0.3), and (18.7 ± 0.3). **(G**) Mean breadth of motor endplate (12.3 ± 0.2), (8.5 ± 0.1), and (11.2 ± 0.2). (**H**) Mean outer radius of motor endplate; (10.7 ± 0.2), (8.7 ± 0.2), and (10.2 ± 0.2). (**I**) Mean inner radius of motor endplate; (1.4 ± 0.05), (1.3 ± 0.04), and (1.6 ± 0.05). (**J**) Mean width of motor endplate depression; (4.9 ± 0.10), (4.8 ± 0.12), and (4.0 ± 0.11). (**K**) Mean depth of motor endplate depression; (2.5 ± 0.05), (1.5 ± 0.04), and (2.2 ± 0.05). (**L**) Mean thickness of left arm of motor endplate; (3.0 ± 0.06), (2.1 ± 0.04), and (2.9 ± 0.05). (**M**) Mean thickness of right arm of motor endplate; (2.8 ± 0.05), (2.0 ± 0.04), and (2.8 ± 0.05). (**N**) Mean thickness of bottom of motor endplate; (3.3 ± 0.06), (2.6 ± 0.05), and (3.4 ± 0.06**)**. (**O**) Mean fluorescence of α-BTX staining; (862.4 ± 23.9), (817.8 ± 22.5), and (793.0 ± 21.8). n is the number of motor endplates from 9–12 mice; *p < 0.05, **p < 0.01, ***p < 0.001, Student’s t-test.

Loss of NAMPT in projection neurons not only impacts the neurons, but also produces changes in the skeletal muscles, most noticeably a significant amount of muscle atrophy^22^. Here, we investigated how the structure of the post-synaptic portion of the NMJ was changed after the deletion of Nampt. To do this, the semitendinosus muscles were labeled with α-BTX to locate the NMJs, embedded in OCT and flash froze in liquid nitrogen. The muscles were then cross-sectioned for imaging. Using Metamorph software, the α-BTX stained region was mapped using an exclusive threshold (Fig. 5A–C). The regions were then subjected to motor endplate measurements (Fig. 5D). Nampt^−/−^ cKO mice had a significantly reduced mean cross-sectional area compared to those in Nampt^f/f^ mice (Fig. 5E). Nampt^−/−^ cKO mice also had significantly shorter mean length, breadth, and outer radius compared to Nampt^f/f^ mice (Fig. 5F–H). The mean inner radius and the width of the endplate depression of NMJ was not significantly different between Nampt^−/−^ cKO and Nampt^f/f^ groups (Fig. 5I,J), but the depth of the depression was significantly reduced in the cKO mice (Fig. 5K). The thickness of the endplate (left arm, right arm, and bottom) was also reduced in the cKO mice compared to those in Nampt^f/f^ mice (Fig. 5L–N).

When the cKO mice were treated with NMN, there were significant restorations of NMJ structure. The mean cross-sectional area of the NMN-treated Nampt^−/−^ cKO mice was significantly larger than Nampt^−/−^ cKO mice but was still less than Nampt^f/f^ mice (Fig. 5E). Mean length, mean breadth, and mean outer radius were significantly longer in the NMN-treated Nampt^−/−^ cKO mice compared to Nampt^−/−^ cKO mice but still significantly reduced compared to Nampt^f/f^ mice (Fig. 5F–H). The mean inner radius for NMN-treated Nampt^−/−^ cKO mice was significantly larger than both Nampt^−/−^ cKO and Nampt^f/f^ mice (Fig. 5I). NMN treatment lead to a significantly narrower depression compared to both Nampt^−/−^ cKO and Nampt^f/f^ mice (Fig. 5J). The NMN treatment also produced a deeper depression than Nampt^−/−^ cKO mice but not as deep as the Nampt^f/f^ mice (Fig. 5K). The thicknesses of the left arm, right arm, and bottom of the motor endplate for the NMN-treated cKO mice were significantly larger than Nampt^−/−^ cKO mice and not different from Nampt^f/f^ mice (Fig. 5L–N). The mean fluorescent intensity of the α-BTX staining, reflecting nicotinic acetylcholine receptor (nAChR) density, of the endplate was only significantly different between the Nampt^f/f^ and NMN-treated Nampt^−/−^ cKO groups (Fig. 5O).

Overall, these data suggest that loss of NAMPT produces post-synaptic structural alterations of the NMJs. These endplates tend to be smaller, flatter and less thick compared to the endplates of Nampt^f/f^ mice, and the treatment of the Nampt^−/−^ cKO mice with NMN prevented most alterations observed. The density of the postsynaptic nAChRs, however, is altered from loss of NAMPT.

### Loss of NAMPT in projection neurons alters skeletal muscle contractile responses

Disabled synaptic transmission in the motor terminal at the NMJ has been suggested to affect muscle function^35,36^. To determine whether Nampt deletion, which impairs synaptic transmission, produces any changes to muscle contraction, semitendinosus muscles were isolated in the same manner as for the previous experiments. In a sylgard coated dish, the wide end of the muscle was pinned down and the other end was connected to a force transducer by a piece of string. The muscle was stimulated at different frequencies and the contractile responses were recorded.

**Figure 6:**
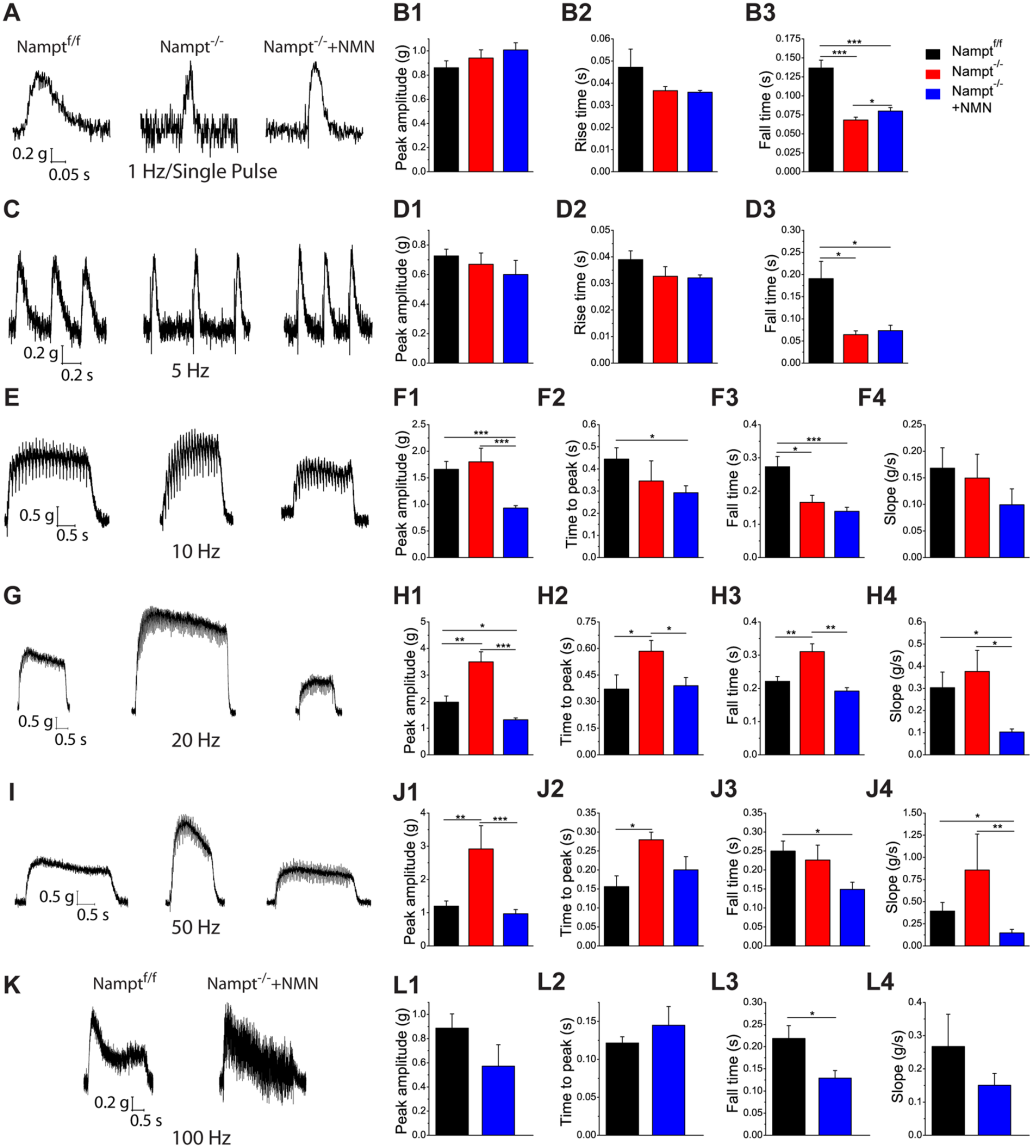
Nampt^−/−^ cKO mice exhibit quicker muscle relaxation and enhanced contractile force but reduced range of responsiveness. **(A**) Representative trace for 1 Hz/Single Pulse stimulation for Nampt^f/f^ (N = 5; n = 57), Nampt^−/−^ cKO (N = 4; n = 53), and NMN-treated Nampt^−/−^ cKO (N = 5; n = 49) mice. (**B**) Mean contractile force (B1), Mean rise time to peak contraction force (B2), and Mean time return to baseline (B3) for 1 Hz/Single pulse stimulation. (**C**) Representative traces for 5 Hz stimulation for Nampt^f/f^ (N = 3; n = 19), Nampt^−/−^ cKO (N = 3; n = 10), and NMN-treated Nampt^−/−^ cKO (N = 2; n = 10) mice. (**D**) Mean contractile force (D1), Mean rise time to peak contraction force (D2), and Mean time return to baseline (D3) for 5 Hz stimulation. (**E**) Representative trace for 10 Hz stimulation for Nampt^f/f^ (N = 4; n = 20), Nampt^−/−^ cKO (N = 4; n = 13), and NMN-treated Nampt^−/−^ cKO (N = 5; n = 22) mice. (**F**) Mean maximum contractile force (F1), Mean time to maximum contraction force (F2), Mean time to return to baseline after stimulation was ended (F3), and Mean slope (in grams force lost per second; calculated from time when maximum contractile force is reached to time when stimulation was removed) (F4) for 10 Hz stimulation. (**G**) Representative trace for 20 Hz stimulation for Nampt^f/f^ (N = 3; n = 9), Nampt^−/−^ cKO (N = 3; n = 10), and NMN-treated Nampt^−/−^ cKO (N = 2; n = 6) mice. (**H**) Mean maximum contractile force (H1), Mean time to maximum contraction force (H2), Mean time to return to baseline after stimulation was ended (H3), and Mean slope (H4) for 20 Hz stimulation. (**I**) Representative trace for 50 Hz stimulation for Nampt^f/f^ (N = 4; n = 18), Nampt^−/−^ cKO (N = 3; n = 5), and NMN-treated Nampt^−/−^ cKO (N = 5; n = 14) mice. (**J**) Mean maximum contractile force (J1), Mean time to maximum contraction force (J2), Mean time to return to baseline after stimulation was ended (J3), and Mean slope (J4) for 50 Hz stimulation. (**K**) Representative trace for 100 Hz stimulation for Nampt^f/f^ (N = 3; n = 8) and NMN-treated Nampt^−/−^ cKO (N = 3; n = 6) mice. (**L**) Mean maximum contractile force (L1), Mean time to maximum contraction force (L2), Mean time to return to baseline after stimulation was ended (L3), and Mean slope (L4) for 100 Hz stimulation. N is the number of mice and n is the number of responses; *p < 0.05; **p < 0.01; ***p < 0.001, Student’s t-test.

The contractile responses can be divided into three patterns based on stimulation frequency: low frequency (1 Hz and 5 Hz), moderate frequency (10, 20, and 50 Hz), and high frequency (100 Hz). For low frequency stimulations, the contractile response patterns were very similar between 1 Hz and 5 Hz. At 1 and 5 Hz stimulation (Fig. 6A,C), peak contractile forces were not significantly different among the three groups (Fig. 6B1,D1). Also, the time to peak force was not significantly different between the three groups (Fig. 6B2,D2). For both frequencies, Nampt^f/f^ mice had a prolonged return to baseline time relative to Nampt^−/−^ cKO and NMN-treated Nampt^−/−^ cKO mice (Fig. 6B3,D3). The return time to baseline for Nampt^−/−^ cKO and NMN-treated Nampt^−/−^ cKO mice was only different at 1 Hz (Fig. 6B3). Overall, these results indicate that skeletal muscles have shorter relaxation times during low frequency stimulation after Nampt deletion.

The response pattern for moderate frequency stimulation is very different from that for low frequencies (Fig. 6E,G,I). The peak contractile forces for Nampt^−/−^ cKO mice were significantly larger than for NMN-treated Nampt^−/−^ cKO mice at 10, 20, and 50 Hz (Fig. 6F1,H1,J1). Nampt^f/f^ and Nampt^−/−^ cKO mice were not different at 10 Hz but Nampt^f/f^ mice produced significantly weaker contractions at 20 and 50 Hz (Fig. 6H1,J1). Nampt^f/f^ mice and NMN-treated Nampt^−/−^ cKO mice had a significant difference in peak contractile force at 10 Hz and 20 Hz but not 50 Hz (Fig. 6F1,H1,J1). Nampt^f/f^ mice had a longer time to reach peak force at 10 Hz (Fig. 6F2). Nampt^−/−^ cKO mice displayed increased time to peak force at 20 and 50 Hz (Fig. 5H2,J2). Nampt^f/f^ mice had a longer return time to baseline at 10 Hz and 50 Hz but Nampt^−/−^ cKO mice had a longer return time at 20 Hz (Fig. 6F3,H3,I3). There was no difference in the slope among the three groups at 10 Hz (Fig. 6F4). For 20 and 50 Hz, NMN-treated Nampt^−/−^ cKO mice had significantly smaller slopes than Nampt^f/f^ mice and Nampt^−/−^ cKO mice (Fig. 6H4,I4). Data from moderate frequency stimulation suggest that loss of NAMPT in the projection neurons may induce significant changes in the muscle, with the muscles attempting to compensate for the loss with increased contractile force. Interestingly, NMN treatment produced a unique phenotype different from Nampt^f/f^ mice and Nampt^−/−^ cKO mice, where the muscles had reduced contractile force but lost force more slowly.

Stimulation with a high frequency (100 Hz) formed a third pattern of response (Fig. 6K). The Nampt^−/−^ cKO mice were completely unresponsive to this stimulation. No significant difference was found in peak contractile force or time to reach peak force between the Nampt^f/f^ and NMN-treated Nampt^−/−^ cKO mice (Fig. 6L1-2). NMN-treated Nampt^−/−^ cKO mice did have a significantly shorter return time to baseline than Nampt^f/f^ mice (Fig. 6L3). There was no significant difference in slope between the two groups (Fig. 6L4). It is noticeable that muscle contractile traces of Nampt^f/f^ mice were smooth, while the muscle contractile traces of NMN-treated Nampt^−/−^ cKO mice exhibited vacillation, indicating a struggle to maintain a muscle contraction. The difference found in the return time to baseline would support this.

Overall, these results indicate changes within the skeletal muscles after Nampt deletion in the projection neurons. The NMN administration seemed to produce an intermediate response state, where the muscle is not unhealthy enough for compensation to occur but also not healthy enough to respond as the Nampt^f/f^ mice do. Treatment with NMN does allow the nerve to remain responsive to high frequency (100 Hz) stimulation, though the response of the muscle suggests some impairment.

### Loss of NAMPT in projection neurons causes sarcomere misalignment and mitochondria morphological change in semitendinosus muscle

**Figure 7:**
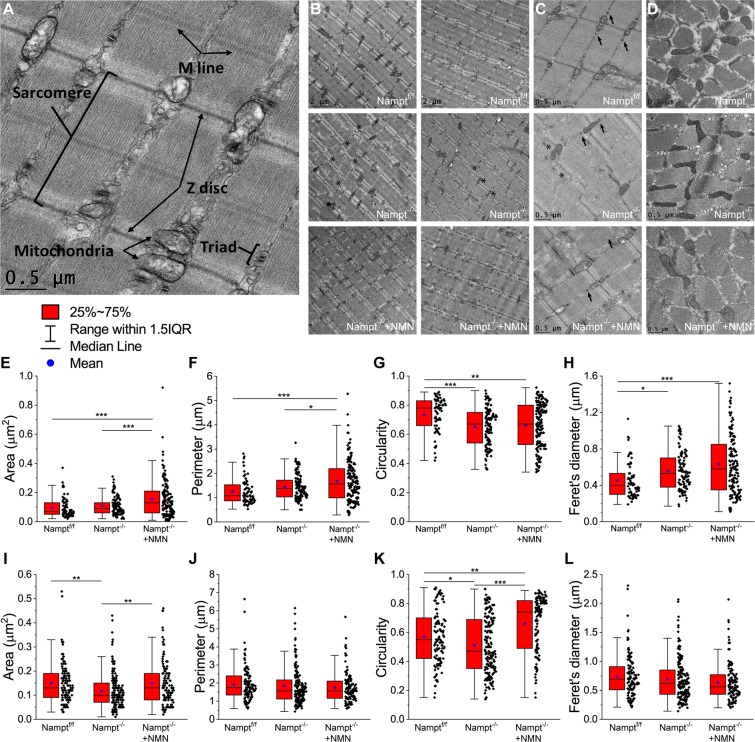
Altered sarcomere alignment and mitochondrial morphology in longitudinal and transverse plane of Nampt^−/−^ cKO mice revealed by TEM. **(A**) Diagram of skeletal muscle components analyzed from TEM images. (**B,C**) Representative longitudinal TEM images of Nampt^f/f^, Nampt^−/−^ cKO, and NMN-treated Nampt^−/−^ cKO mice. Arrows indicate triadic junctions and asterisks indicate misaligned Z discs. (**D**) Representative transverse TEM images of Nampt^f/f^, Nampt^−/−^ cKO, and NMN-treated Nampt^−/−^ cKO mice. (**E**) Mitochondrial area in longitudinal plane from Nampt^f/f^ (n = 65), Nampt^−/−^ cKO (n = 109), and NMN-treated Nampt^−/−^ cKO (n = 140) mice. (**F**) Mitochondrial perimeter in longitudinal plane from Nampt^f/f^, Nampt^−/−^ cKO, and NMN-treated Nampt^−/−^ cKO mice. (**G**) Mitochondrial circularity in longitudinal plane from Nampt^f/f^, Nampt^−/−^ cKO, and NMN-treated Nampt^−/−^ cKO mice. (**H**) Mitochondrial perimeter in longitudinal plane from Nampt^f/f^, Nampt^−/−^ cKO, and NMN-treated Nampt^−/−^ cKO mice. (**I**) Mitochondrial area in transverse plane from Nampt^f/f^ (n = 115), Nampt^−/−^ cKO (n = 170), and NMN-treated Nampt^−/−^ cKO (n = 118) mice. (**J**) Mitochondrial perimeter in transverse plane from Nampt^f/f^, Nampt^−/−^ cKO, and NMN-treated Nampt^−/−^ cKO mice. (**K**) Mitochondrial circularity in transverse plane from Nampt^f/f^, Nampt^−/−^ cKO, and NMN-treated Nampt^−/−^ cKO mice. (**L**) Mitochondrial perimeter in transverse plane from Nampt^f/f^, Nampt^−/−^ cKO, and NMN-treated Nampt^−/−^ cKO mice. n is the number of mitochondria from 1–2 mice; *p < 0.05, **p < 0.01, ***p < 0.001 ANOVA test.

Based on the results from the skeletal muscle contractile response, we decided to investigate whether loss of NAMPT may also produce changes to skeletal muscle structure and mitochondria morphology in conjunction to the functional changes. The mitochondria in skeletal muscles can be divided into two groups, the sub-sarcolemmal (SS) and the intermyofibrillar (IMF). The SS and IMF mitochondria differ in location, morphology, and physiological properties^32,37,38^. IMF mitochondria are also thought to be more important for muscle contraction^37,39^. To investigate how IMF mitochondrial morphology may be affected following Nampt deletion, we performed TEM on semitendinosus muscles dissected from Nampt^−/−^ cKO and NMN-treated Nampt^−/−^ cKO 23 days after the final TAM injection, as well as aged matched Nampt^f/f^, mice to assess myofiber composition and mitochondrial morphology. Figure 7A shows the analysis of different structures of a longitudinal section.

Skeletal muscle myofibers are made up of many smaller functional units called sarcomeres. Skeletal muscles from Nampt^f/f^ mice had myofibers with well aligned sarcomeres, with Z discs typically in line with the Z discs of adjacent sarcomeres (Fig. 7B, top panels). Nampt^−/−^ cKO mice displayed noticeable sarcomere misalignment, with some areas being well aligned but other areas having no alignment (Fig. 7B, middle panels, asterisks). NMN-treated Nampt^−/−^ cKO mice had sarcomere alignment similar to the Nampt^f/f^ mice (Fig. 7B, bottom panels). There did not seem to have any noticeable changes in the triadic junctions, a group composed of a transverse tubule flanked by sarcoplasmic reticulum, in the Nampt^−/−^ cKO or NMN-treated Nampt^−/−^ cKO mice (Fig. 7C, arrows). Thus, deletion of Nampt causes considerable misalignment of sarcomeres which can be restored by NMN treatment.

Longitudinal (Fig. 7C) and transverse (Fig. 7D) mitochondrial morphology was altered for both Nampt^−/−^ cKO and NMN-treated Nampt^−/−^ cKO mice. Longitudinal mitochondrial area was significantly larger for NMN-treated Nampt^−/−^ cKO mice (Fig. 7E). The perimeter was also significantly longer for NMN-treated Nampt^−/−^ cKO mice (Fig. 7F). Namp^−/−^ cKO and Nampt^f/f^ mice did not differ for area or perimeter. Circularity was significantly lower for both Nampt^−/−^ cKO and NMN-treated Nampt^−/−^ cKO mice (Fig. 7G). Feret’s diameter was significantly longer in Nampt^−/−^ cKO and NMN-treated Nampt^−/−^ cKO mice (Fig. 7H). Transverse mitochondrial area was reduced in the Nampt^−/−^ cKO mice (Fig. 7I). Though, no difference was observed in perimeter length among the three groups (Fig. 7J). Circularity was significantly higher in the NMN-treated Nampt^−/−^ cKO mice and significantly lower in the untreated Nampt^−/−^ cKO mice (Fig. 7K). No difference was observed in Feret’s diameter (Fig. 7L). Overall, these results suggest that deletion of Nampt alters skeletal muscle mitochondria morphology, which cannot be restored by NMN treatment.

## Discussion

The results from the current study demonstrate how loss of NAMPT in projection neurons produces detrimental effects on vesicle cycling, endplate morphology, and muscle fiber structure and function using semitendinosus muscle preparations. Neurons lacking NAMPT displayed impaired endocytosis and exocytosis based on live muscle imaging using FM1-43 dye. Both the rate of dye uptake/release and total amount of dye loaded/unloaded were reduced in the Nampt^−/−^ cKO mice. Our data also suggests that Nampt^−/−^ cKO mice are unable to mobilize all labeled synaptic vesicles. Spontaneous endocytosis was unaffected by the loss of NAMPT. The detrimental effects observed in Nampt^−/−^ cKO mice were alleviated when the NAD^+^ precursor, NMN, was administrated for 2 weeks. It is important to note that there was no reduction in iNAMPT or eNAMPT levels in semitendinosus muscle in the cKO mice and there was no increase in NAD^+^ levels in semitendinosus muscle following NMN injection in control mice, which is consistent with results from prior studies^9,22^.

The reduction in total FM1-43 fluorescence increase and decrease after stimulation in the Nampt^−/−^ cKO mice could be explained by a reduction of synaptic vesicle number, which has been observed in the NMJs of different gene deletion and mouse disease models that have exhibited synaptic transmission dysfunction^40–42^. Previous experiments in Nampt^−/−^ cKO mice has also supported the possibility of a smaller pool of vesicles^22^. However, this would not fully explain our results because fewer synaptic vesicles should result in faster saturation/depletion of the fluorescence. Our results show that Nampt^−/−^ cKO mice were slower loading and unloading FM1-43, suggesting a malfunction of the synaptic vesicle recycling in projection neurons. The vesicles used in synaptic signaling can be separated into two different pools: the readily-releasable pool (RRP) and the reserve pool (RP)^43^. Stimulation at 10 Hz is believed to load FM1-43 into vesicles of both pools^44^, so our experiments should be labeling vesicles in the RRP and RP. Identifying where the problem is occurring may be challenging because there are many proteins involved in synaptic vesicle recycling^45^. Alterations to Ca^2+^ levels could also be occurring, given the critical role of Ca^2+^ has in endocytosis and exocytosis^46,47^.

The results from our initial destaining data led us to investigate whether Nampt^−/−^ cKO mice had problems releasing certain FM1-43 loaded vesicles or whether the rate of exocytosis was reduced. If only the rate of exocytosis had been affected, then increasing the duration of destaining stimulation would provide more time for the FM1-43 labeled vesicles to be released and for fluorescence to steadily decline. On the other hand, if certain labeled vesicles cannot be released, then the fluorescence decrease would stop at some timepoint and the intensity level remain relatively stable. We observed the latter, indicating that Nampt^−/−^ cKO mice may not be able to access certain synaptic vesicles, possibly a result of RP vesicles not being moved to the RRP. The specific mechanisms that mobilize a synaptic vesicle from the RP to the RRP are not well understood^43^. Our results are similar to the differences observed in 10 Hz vs. 100 Hz exocytosis^44^, suggesting that Nampt deletion may inappropriately activate rapid recycling of vesicles at lower frequencies. Given the importance of NAMPT activity in energy metabolism and homeostasis^1,2,4^, Nampt deletion in projection neurons, which depletes NAD^+^ and disrupts bioenergetic homeostasis^22^, may also cause a disruption of the synaptic vesicle cycling during stimulation due to a lack of energy. A recent study showed that NMNAT, the enzyme immediately after NAMPT and converts NMN into NAD^+^, can impact synaptic transmission^34^, providing evidence that alteration of bioenergetics through NAD^+^ salvage pathway perturbation may directly influence synaptic vesicle cycling.

A significant alteration in the morphology of the motor endplate was observed in Nampt^−/−^ cKO mice. Loss of NAMPT produced a smaller, flatter, and thinner motor endplate but receptor density did not seem to be affected. The clustering and recycling of the nAChRs, which is an important aspect of normal NMJ maintenance, has been a focus of a large amount of research^48^. Our results do not suggest any problems with the localization of nAChRs, indicating that the signaling pathway and microtubules involved in clustering and inserting nAChRs may not have been affected in the cKO mice^49^. Future study should focus on proteins involved in maintenance of NMJ structure as well as proteins involved in nAChR expression and formation.

Our results from the muscle contraction experiment were unexpected. After low frequency stimulation (1 Hz or 5 Hz), we observed no change in peak force and rise time in Nampt^−/−^ cKO mice. The return time to baseline, indicating muscle relaxation, was significantly faster in both Nampt^−/−^ cKO conditions. This suggests that the speed or amount of Ca^2+^ released has not been affected but that Ca^2+^ may be removed from the cytosol faster. A possible explanation for our results could be upregulation of SERCA expression in the Nampt^−/−^ cKO mice. The sarcoplasmic reticulum (SR) is the main storage site of Ca^2+^ in muscle and the sarco-(endo) plasmic reticulum Ca^2+^-ATPase (SERCA) is responsible for clearing the Ca^2+^ from the cytosol. The impact of the deletion of Nampt became more evident as the stimulation frequency was increased. Loss of NAMPT appears to induce a compensatory response in the skeletal muscle, where Nampt^−/−^ cKO mice exhibit enhanced contractile force across moderate stimulation frequencies. Providing NMN to the Nampt^−/−^ cKO mice did not restore the normal contractile response, but instead produced a unique phenotype. This was surprising given the results indicating how NMN administration can return synaptic function and motor endplate morphology to relatively normal levels. It is possible that the supplementation produced partial recovery. NMN may have prevented the downstream effects that resulted in the increased contractile force observed in the Nampt^−/−^ cKO mice but was insufficient for maintaining normal muscle contraction. Instead, the NMN-treated mice had responses that resembled the Nampt^f/f^ mice in shape but with a reduced force.

Excitation-contraction coupling has been well defined in skeletal muscles and there are many possibilities where changes could have occurred^50^. Altered Ca^2+^ clearance could also be responsible, Nampt^−/−^ cKO mice may experience increased contractile force due to elevated Ca^2+^ over moderate stimulation frequencies. Investigation of Ca^2+^ movement and the excitation-contraction pathway is needed. The lack of response to 100 Hz stimulation in Nampt^−/−^ cKO mice is consistent with previous research showing motor nerves lacking NAMPT exhibited impaired synaptic transmission at high stimulation frequencies^22^. Direct stimulation of the muscle would be needed to determine the contractile response of muscles from Nampt^−/−^ cKO mice. NMN treatment allowed the motor nerves to respond to 100 Hz stimulation but the muscle contractions still indicated some impairment compared to Nampt^f/f^ mice.

We also observed changes to myofiber arrangement and mitochondrial morphology. Sarcomere misalignment was common in Nampt^−/−^ cKO mice, which can be prevented by NMN treatment. Sarcomere misalignment has been observed in animal models for muscular dystrophy, ataxia telangiectasia, and amyotrophic lateral sclerosis (ALS), as well as in denervated muscles and indicates changes in normal myofiber interactions^51–54^. We also observed no differences in the triadic junction. Alterations to triadic junctions has been observed in skeletal muscle-specific NAMPT knockout mice, as well as in denervated rat skeletal muscles^9,53^. Nampt^−/−^ cKO mice undergo significant denervation^22^ so the lack of triad alterations is slightly unexpected. However, this could be due to the short life span of our mice following Nampt deletion. The mice typically will not survive beyond 4 weeks following TAM injection, while the triad alterations observed in the skeletal muscle NAMPT knockout mice were observed at 7 months of age and from rats that had been denervated for 56 days^9,53^. In could simply be that Nampt^−/−^ cKO mice do not survive long enough for these alterations to occur.

IMF mitochondrial morphology is significantly different following the loss of NAMPT. The mitochondria from Nampt^−/−^ cKO mice were similar, if not slightly smaller, in size relative to Nampt^f/f^ mice but were abnormally shaped. The mitochondria from NMN-treated Nampt^−/−^ cKO mice were also abnormally shaped but also seemed to larger, especially compared the untreated Nampt^−/−^ cKO mice. No swelling or cristae disruptions were observed in any condition, which are common for mitochondrial dysfunction^55^. It is widely thought that mitochondrial dysfunction and abnormal mitochondrial morphology are related. This is supported from experimentation in aged and disease models of mice^9,32,54–56^. We did not perform any functional experimentation on skeletal muscle mitochondria but Nampt^−/−^ cKO and NMN-treated Nampt^−/−^ cKO mice both demonstrated abnormal mitochondria morphology relative to Nampt^f/f^ mice, so possible functional changes would not be unexpected.

In summary, the current study found that deletion of Nampt from projection neurons causes detrimental effects on NMJ function, endplate morphology and muscular structure and contractility. Further experiments are needed to identify whether these results are the product of very specific alterations or caused by changes in normal neuronal function. As the symptoms (*e.g*., muscle atrophy, paralysis and NMJ dysfunction) of the Nampt^−/−^ cKO mice resemble those in ALS model and NAMPT is downregulated in human ALS brain^22^, the current study provides novel insights for the involvement of the NAMPT-mediated NAD^+^ salvage pathway in the pathology of ALS. The current study also suggests that NMN may be a useful therapeutic agent for skeletal muscle diseases.

## Supplementary information

Supplementary Figure 1.

## Data Availability

The datasets generated during and/or analyzed during the current study are available from the corresponding author on reasonable request.
