# Supplementary material for: The effect of NAMPT deletion in projection neurons on the function and structure of neuromuscular junction (NMJ) in mice
Source: Sci Rep. 2020 Jan 9;10:99. doi: 10.1038/s41598-019-57085-4 (PMC6952356; doi:10.1038/s41598-019-57085-4)
Supplement: Supplementary file 1 — Supplementary Figure 1. [file 41598_2019_57085_MOESM1_ESM.pdf]

The effect of NAMPT deletion in projection neurons on the function and structure of neuromuscular junction (NMJ) in mice

Samuel Lundt[1], Nannan Zhang[2], Xiaowan Wang[1], Luis Polo-Parada[2,3], Shinghua Ding[1,2*]

[1]Dept. of Biomedical, Biological and Chemical Engineering

[2]Dalton Cardiovascular Research Center

[3]Dept. of Medical Pharmacology and Physiology

University of Missouri, Columbia, MO 65211

[*]Corresponding author: Shinghua Ding, Dalton Cardiovascular Research Center, Dept. of Biomedical, Biological and Chemical Engineering, University of Missouri-Columbia, 134 Research Park Drive, Columbia, MO 65211. E-mail:dings@missouri.edu. Tel: 573 884 2489.

Supplementary Figures

A

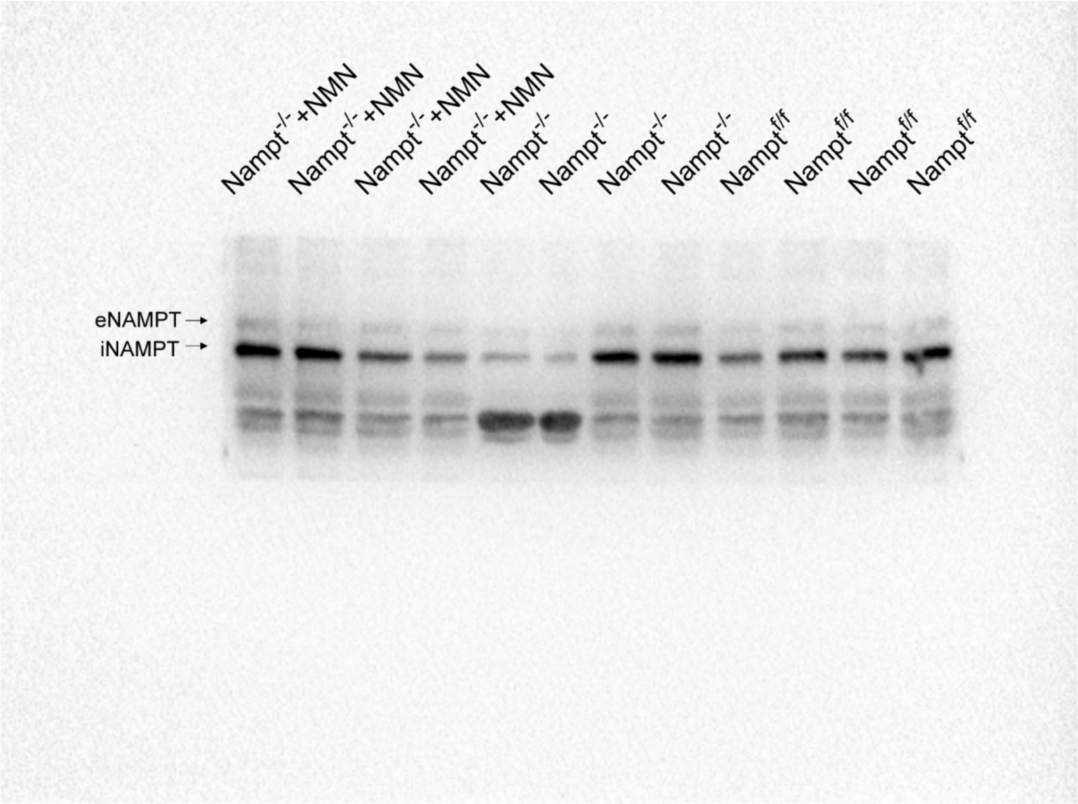

B

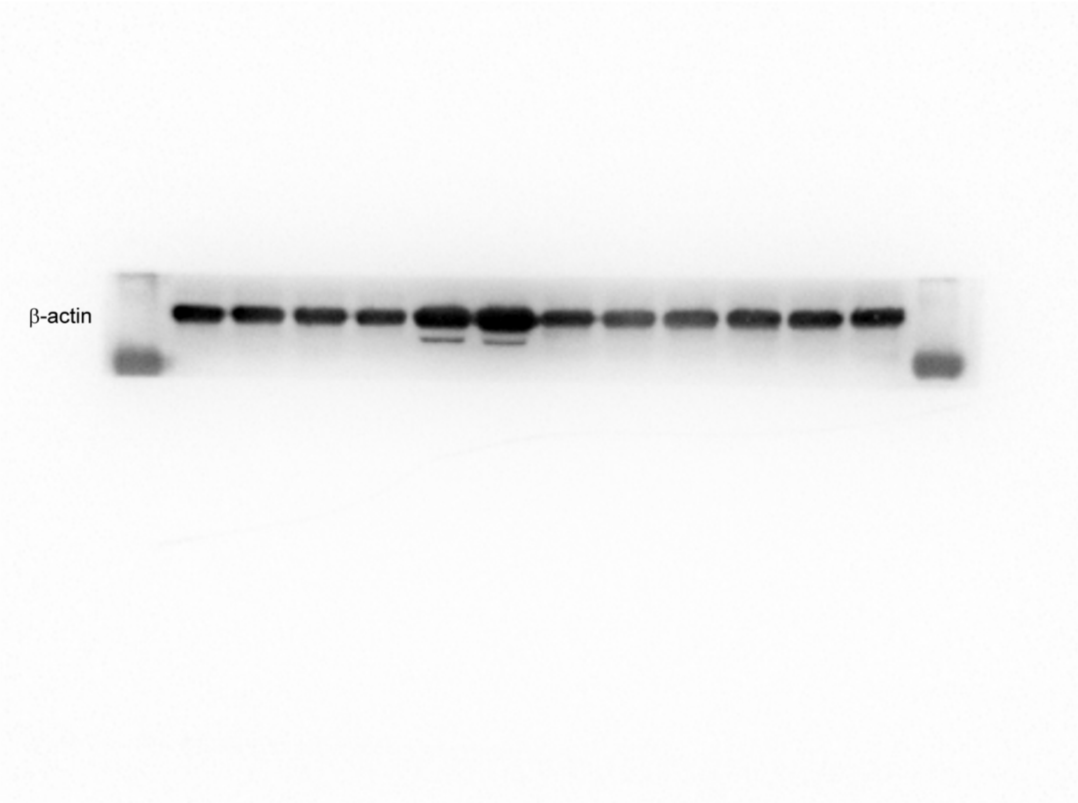

Supplemental Figure 1. Full Western blotting images of NAMPT levels in semitendinosus muscle from Nampt$^{f/f}$, Nampt$^{-/-}$, and Nampt$^{-/-}$ +NMN mice. A) Original image of film incubated with anti-NAMPT antibody (Exposure time=7.9 seconds). B) Original image of film incubated with anti-β-actin (Exposure time=1.0 seconds). Images are from different gels that were ran with the same sample order at the same time.
